# Plant-Derived Anti-TMV Metabolites: Mechanisms, Limitations and Future Perspectives

**DOI:** 10.3390/v18070756

**Published:** 2026-07-09

**Authors:** Muhammad Qasim Aslam, Ziran Gao, Amr Said Mohamed, Samah Mostafa El-Sayed, Wenjing Yang, Lin Cheng, Kuo Wu, Yu Li, Yongdui Chen

**Affiliations:** 1 Biotechnology and Germplasm Resources Institute, Yunnan Academy of Agricultural Sciences/Yunnan Provincial Key Laboratory of Agricultural Biotechnology/Key Lab of Southwestern Crop Gene Resource and Germplasm Innovation, Ministry of Agriculture and Rural Affairs, Kunming 605205, China; qasim2893@gmail.com (M.Q.A.); 13013459139@163.com (Z.G.); amr_amz_9@yahoo.com (A.S.M.);; 2 Faisalabad Campus, Department of Biotechnology, Faculty of Rehabilitation and Allied Health Sciences, Riphah International University, Faisalabad 38070, Pakistan; 3 School of Ethnic Medicine, Yunnan Minzu University, Kunming 650504, China; 4 Botanical Gardens Research Department, Horticulture Research Institute of Agricultural Research Center, Giza 12513, Egypt; 5 Ornamental Plants and Woody Trees Department, Agricultural and Biological Research Institute, National Research Centre, Giza 12513, Egypt; ensamah_83@hotmail.com

**Keywords:** tobacco mosaic virus, plant-derived compounds, anti-TMV activity, antiviral mechanisms, coat protein and induce resistance

## Abstract

Tobacco mosaic virus (TMV) poses a serious threat to global agricultural production. It is an exceptionally stable virus with a broad host range and is widespread across diverse agroecosystems. Concerning TMV management, plant-derived metabolites have emerged as promising and eco-friendly antiviral agents. To date, numerous plant-derived metabolites with potent anti-TMV activity and their underlying mechanisms of action have been identified. However, a comprehensive understanding of their mechanisms of action is still lacking. This review summarizes the diversity of anti-TMV mechanisms triggered by natural and plant-sourced semisynthetic compounds. These metabolites mainly include alkaloids, flavonoids, terpenoids, phenylpropanoids, and glycosides, which act either directly targeting virus particles or indirectly by eliciting host immunity. Together, these mechanisms form an integrated defence network that restricts viral replication and movement within the host. This mechanistic understanding will be essential for the rational development of sustainable and effective plant-derived antiviral agents.

## 1. Introduction

Plant viruses pose a serious threat to global agricultural production due to the difficulty of effective control measures [1]. As obligate intracellular pathogens, viruses disrupt key metabolic processes in plant cells, leading to stunted growth, reduced photosynthetic efficiency, and compromised crop yield and quality [2]. Precise estimates of their economic impact remain elusive; however global losses inflicted by viral diseases are estimated to exceed $30 billion annually [2,3]. Tobacco Mosaic Virus (TMV) is one of the earliest known and most studied plant pathogens [4,5]. It infects more than 885 plant species, including tobacco, tomato, pepper, cucumber, and numerous ornamental plants [6]. Its highly stable virion structure contributes to prolonged infectivity and widespread distribution across diverse agroecological regions [1]. The annual economic loss caused by TMV alone is around $100 million [2,4]. From 2013 to 2017, mainland China conducted a comprehensive survey on the diversity of plant viruses infecting vegetable crops across 31 provinces. The survey encompassed more than 41,000 vegetable samples from the Solanaceae, Cucurbitaceae, Leguminosae, and Cruciferae families and identified 63 viruses in all studied provinces, where TMV appeared as the dominant virus in Solanaceae, Cucurbitaceae, and Cruciferae vegetable crops [7].

Concerning TMV threat management, implications of resistance (R) genes have long been considered a key strategy for controlling TMV infection in crop plants. R gene-mediated resistance is highly specific, relying on the recognition of particular viral effectors by host-encoded resistance proteins. Consequently, different plant species have evolved distinct resistance genes that confer protection against TMV through diverse recognition and defence activation mechanisms. For example, in tomato, well-characterised resistance genes, *Tm-1*, *Tm-2*, and *Tm-2^2^*, have been widely utilised, either individually or in combination, to confer protection against TMV [8,9,10]. Likewise, leucine-rich repeat (LRR)-containing *L* genes offer resistance in pepper against TMV [1] and *N* gene—introgressed from wild tobacco relatives—provides resistance against TMV by inducing hypersensitive response mechanisms [11]. However, despite its effectiveness, *N*-gene-mediated resistance has not been extensively deployed in commercial tobacco cultivars [12]. Moreover, although R gene-mediated resistance provides highly specific and effective protection against targeted viral strains, its durability is often limited. The rapid evolution and genetic variability of viruses enable the emergence of mutant strains capable of bypassing host recognition, thereby compromising the R gene-mediated resistance over time [9].

Presently, the management of plant viral diseases largely relies on chemical control strategies, including pesticides that suppress insect vectors and antiviral compounds that interfere with viral replication or infection processes [13,14]. However, this approach raises safety concerns, including adverse effects on non-target organisms, degradation of soil and water quality, and potential risks to human well-being [15]. For example, the two famous pesticides, i.e., organophosphates and dichlorodiphenyltrichloroethane, launched in the early 1930s, are now prohibited in most agricultural countries due to their deleterious effects on human health and other animals. Moreover, the continued use of chemical pesticides can promote the development of pest resistance; thus, over time, these potent drugs lose their efficacy [15,16]. Meanwhile, plant-derived natural compounds have emerged as promising alternatives for sustainable agricultural production. Their eco-friendly nature offers advantages such as biodegradability, minimal off-target effects, and a reduced likelihood of resistance development in target pathogens [17].

Current research on plant-derived metabolites with anti-TMV potential has evolved significantly over the past few decades [18]. Early studies focused on identifying antiviral compounds, followed by their isolation and characterization through in vivo assays [19,20]. This gradually progressed towards understanding their modes of action, underlying molecular mechanisms, and roles in immune response pathways [21,22]. Subsequent advances emphasised structure–activity relationship studies and the synthesis of novel derivatives with improved antiviral efficacy [23,24]. The recent integration of multi-omics approaches has enabled deeper insights into the regulation of immune pathways and host-mediated systemic acquired resistance (SAR) responses [25]. To date, hundreds of plant-derived metabolites have been reported with significant anti-TMV efficacy, yet fewer studies have detailed their potential antiviral mechanisms [18], although understanding these mechanisms is crucial for supporting the development of eco-friendly and sustainable plant-based antiviral agents. This review aims to consolidate current knowledge on anti-TMV mechanisms of plant-derived metabolites, along with selected semi-synthetic and plant-inspired compounds, providing a detailed overview of the diverse pathways through which these compounds exert anti-TMV activity.

## 2. Biology of TMV

**Figure 1:**
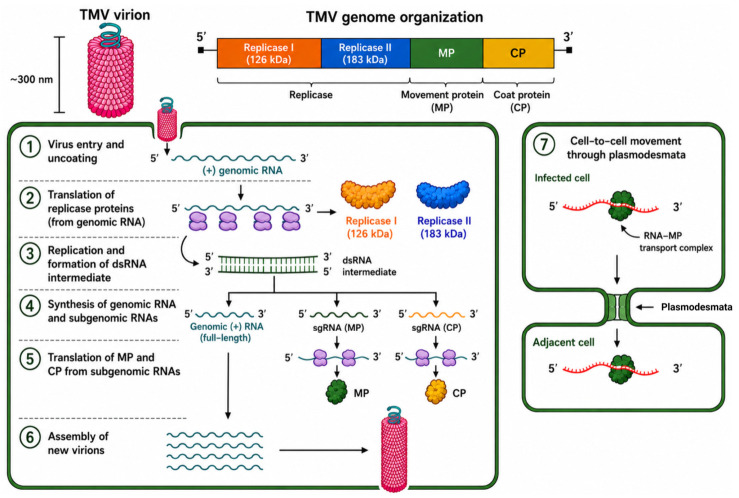
Schematic representation of the structure of TMV, genome organisation, and replication cycle in a plant cell. Following entry into the host cell, the viral positive-sense single-stranded RNA [(+) genomic RNA] is uncoated and directly translated to produce replication-associated proteins (size 126 kDa and 183 kDa). Viral replication proceeds through the formation of an intermediate double-stranded RNA (dsRNA), which serves as a template for the synthesis of progeny viral RNA molecules named subgenomic RNA (sgRNA) and viral genomic RNA. The sgRNA molecules served as a template for the synthesis of viral movement (MPs) and coat 7CPs). The newly synthesised viral genomic RNAs and CP molecules assemble into mature virions, which subsequently move through plasmodesmata to neighbouring cells with the help of MP complex. The picture was revised in BioRender https://BioRender.com (accessed on 6 July 2026). Created in BioRender. Siddiqui, H. (2026) https://BioRender.com/86hl1hy (accessed on 6 July 2026).

TMV is a member of the genus *Tobamovirus*, family *Virgaviridae*, characterised by a highly stable virion that can persist for extended periods in infected plant debris [26]. It is primarily transmitted through mechanical means, including direct contact, using contaminated tools, and/or by infected plant materials. Although chewing insects may facilitate incidental transfer through contaminated mouthparts, they are not considered true biological vectors of TMV [27]. Regarding TMV structure, Tobamoviral virions are rod-shaped with a rigid helical structure (Figure 1), measuring approximately 300 nm in length and 18 nm in diameter, and possessing a central channel of 4 nm in diameter. The virion encapsidated by approximately 2100 identical coat protein subunits arranged in a right-handed helical configuration. This highly ordered structure contributes to the remarkable environmental stability of TMV, enabling it to remain infectious under a wide range of environmental conditions [28].

The TMV genome is a positive-sense single-stranded RNA molecule of approximately 6.3–6.5 kb, encoding four major proteins [29], two replicase proteins 126 kDa and 183 kDa, which are primarily involved in viral RNA replication. The 183 kDa protein is produced via read-through of the 126 kDa open reading frame and contains core RNA-dependent RNA polymerase (RdRp) activity [30]. The movement protein (~30 kDa) facilitates the cell-to-cell movement of viral RNA through plasmodesmata, while the coat protein (CP) (~17.5 kDa) is responsible for RNA encapsidation and is essential for long-distance systemic movement within the host [31,32,33]. Regarding the virus replication cycle, following entry into the host cell, viral RNA acts directly as messenger RNA and is translated to produce replicase proteins. These proteins, together with viral RNA, form replication complexes found associated with host cell membranes, particularly with the endoplasmic reticulum [29,34]. Replication involves the synthesis of a complementary negative-strand RNA intermediate, which serves as a template for the production of new positive-strand viral genomes. The role of sgRNA synthesis in virus replication will be discussed later. The movement protein directs viral RNA to plasmodesmata—the intercellular channels used for cell-to-cell movement, enabling intercellular movement of the viral RNA-protein complexes by increasing the plasmodesmata size exclusion limits (Figure 1) [33]. The CP facilitates long-distance transport via the phloem vascular system, leading to the establishment of systemic infection [35].

TMV infection results in a wide range of symptoms affecting both vegetative and reproductive plant parts. Common symptoms include mosaic patterns, chlorosis, necrotic lesions, leaf curling, vein clearing, and stunted growth. In flowers, infection may cause colour breaking, while in fruits and vegetables, it may cause discolouration, malformation, and reduced quality. In some cases, stem abnormalities such as pitting or grooving may also be observed, reflecting the systemic nature of TMV infection [36].

## 3. Overview of Plant-Derived Anti-TMV Metabolites

Although plants appear sessile and passive, they exhibit highly dynamic biological activity in response to continuous exposure to biotic and abiotic stresses. Plants produce a wide array of biologically active defence metabolites, collectively known as secondary metabolites, to survive under such conditions [3]. These compounds assist plant immunity by directly inhibiting pathogen infection or by modulating plant stress-responsive pathways [6]. In recent years, plant-derived secondary metabolites have attracted considerable attention as potential antiviral agents. Studies published between 2010 and 2025 have identified a wide range of natural and synthetic plant-derived metabolites exhibiting anti-TMV activity, including alkaloids, flavonoids, terpenoids, phenolic compounds, glycosides, and steroidal compounds [18], see Table 1.

Alkaloids are nitrogen-containing compounds widely distributed in plants with their antiviral, antibacterial, antitumor and analgesic effects [65]. Considering their structural diversity, alkaloids are divided into various classes like indole alkaloids, isoquinoline alkaloids, quinoline alkaloids, diterpenoid alkaloids, quinazolinone alkaloids and phenanthroindolizidine alkaloids. These compounds exhibit strong anti-TMV activity and often interfere with viral replication and protein synthesis, thereby reducing viral accumulation in infected tissues [41,44,61].

Flavonoids represent one of the most extensively investigated classes of plant secondary metabolites exhibiting anti-TMV activity. Major subclasses, including flavones, flavonols, isoflavones, and chalcones, have demonstrated significant inhibitory, protective, and, in some cases, curative effects against TMV infection [46,66]. Given their mechanism, these compounds predominantly exert their antiviral activity by modulating host defence responses, including activating defence-related enzymes and upregulating resistance-associated genes, thereby enhancing host-mediated immunity [6,45,46,51,52,56]. However, flavonoids also exert direct antiviral activity in addition to inducing host immunity by interacting with the viral CP, leading to structural destabilisation and reduced viral infectivity [51,52].

Terpenoids constitute a large and structurally diverse class of plant secondary metabolites, characterised by isoprene units as their fundamental building blocks. This group includes monoterpenes, sesquiterpenes, diterpenes, and triterpenes, many of which have demonstrated notable antiviral activity against TMV. Terpenoid compounds isolated from *Tithonia diversifolia* and *Nicotiana tabacum* have shown significant anti-TMV effects either by inhibiting viral replication and assembly [40,41,42] or inducing systemic resistance in the host by activating defence-related pathways [6,25,49].

Phenylpropanoids, including phenylpropanoic acids, coumarins, and lignans, represent an important class of antiviral phenolic compounds. Among these, phenylpropanoic acid derivatives largely exert their antiviral effects by inducing systemic resistance, involving the activation of host defence signalling pathways and associated gene expression [50,57,62]. In contrast, coumarins exhibit direct antiviral effects—such as inhibition of viral replication and accumulation and also induce indirect mechanisms mediated through enhancement of host resistance [6,45]. Lignans have also been observed to contribute to anti-TMV activity, although their mechanisms are comparatively less well characterised.

Steroidal glycosides and other glycosylated compounds have often demonstrated strong antiviral activity at relatively low concentrations. Their mode of action involves suppression of viral protein synthesis by targeting viral subgenomic RNA molecules [37,39] or inhibiting downstream viral protein synthesis [63]. Collectively, these findings suggest that different classes of plant-derived metabolites employ distinct yet overlapping antiviral strategies, which are discussed in detail below.

## 4. Anti-TMV Mechanisms

**Figure 2:**
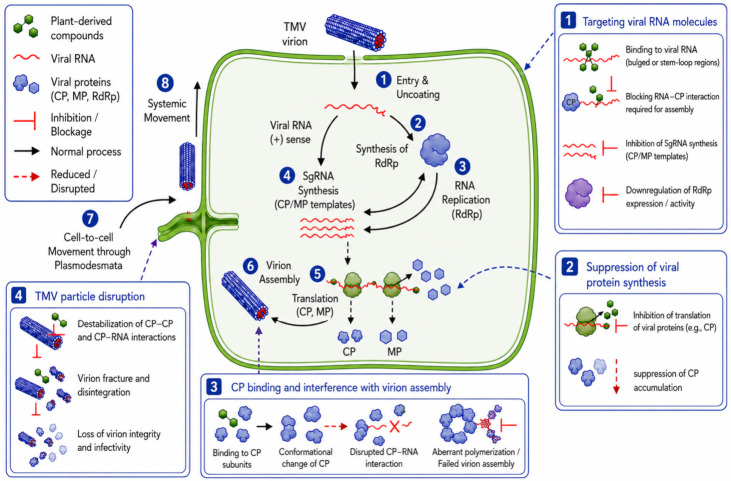
Direct antiviral mechanisms of plant-derived compounds against the TMV infection cycle in plant cells. Plant-derived compounds interfere with various stages of the TMV infection cycle, targeting viral RNA replication and SgRNA synthesis, suppressing CP synthesis, disrupting virion assembly and destabilising the structure of viral particles. The picture was revised in BioRender https://BioRender.com (accessed on 6 July 2026). Created in BioRender. Siddiqui, H. (2026) https://BioRender.com/q5pkaq6 (accessed on 6 July 2026).

Plant-derived compounds exhibit diverse modes of action against TMV infection, as different classes of bioactive metabolites target distinct stages of the viral life cycle. In recent years, increasing attention has been directed toward elucidating the underlying mechanisms of action of these compounds, which is essential for understanding how they interact with viral components and activate host defence systems. Such mechanistic insights are critical not only for effective TMV management but also for the rational development of novel antiviral agents [24,56,57,67]. Current evidence suggests that the antiviral activities of plant-derived metabolites can be broadly categorised into two principal modes: (1) direct interactions with viral components or (2) indirectly inducing host immunity. Direct antiviral mechanisms act by targeting multiple stages of the TMV life cycle, including (i) interference with viral replication, often through targeting viral RNA molecules; (ii) suppression of viral proteins required for virion replication and systemic spread; (iii) disruption of virion assembly; and (iv) alteration or destabilisation of viral CP structure, thereby compromising virion integrity (Figure 2). In contrast, indirect mechanisms involve the induction of systemic acquired resistance (SAR), characterised by the activation of defence-related genes and associated hormonal signalling pathways, particularly salicylic acid- and jasmonic acid-dependent responses, or by inducing ribosome-inactivating proteins (RIPs), which cause structural modifications in host ribosomal RNA molecules and inhibit viral protein synthesis. Overall, these mechanisms disrupt the TMV disease cycle by impeding its replication and systemic movement in the host plant. These mechanisms are discussed in detail below.

### 4.1. Direct Anti-TMV Mechanisms

#### 4.1.1. Targeting Viral RNA Molecules

For rapid onset of viral infection, the virus synthesises short RNA molecules called subgenomic RNAs (sgRNAs), to boost the synthesis of viral proteins [68,69]. Seco-pregnane steroidal glycosides sourced from *Strobilanthes cusia* predominantly inhibit TMV replication and systemic spread by selectively suppressing the synthesis of viral SgRNA molecules, which encode CP and MPs [37]. Another RNA targeting mechanism has been reported for antofine and its synthetic analogues [38]. This mechanism does not target viral SgRNA molecules and CP synthesis; rather, it represents a highly effective strategy to inhibit virion assembly by specifically binding to unpaired bases or bulged structures in TMV genome. These complexes interfere with critical RNA–CP interactions required for nucleation and elongation processes. During virion assembly, CP recognises stem-loop structures in the viral genome [70], By binding to unpaired bases or bulged RNA structures, these small molecules create a physical hindrance that prevents proper encapsidation of the viral genome, thereby inhibiting TMV infection.

Similarly, synthetic derivatives of lycoricidine interfere with TMV replication and viral protein synthesis. The RT-PCR assay and Western blot analyses showed reduced CP transcript levels and protein accumulation [43]. However, the precise mechanism underlying this inhibitory activity remains unclear. Likewise, two sesquiterpenoids, tagitinin C and 1β-methoxydiversifolin-3-O-methyl ether, isolated from *Tithonia diversifolia*, exhibit notable anti-TMV activity by suppressing the transcript levels of CP and the RNA-dependent RNA polymerase (RdRp) enzyme [40]. RdRp plays a vital role in viral RNA synthesis. Therefore, its downregulation indicated partial inhibition of viral replication, whereas reduced CP levels limited virion assembly and accumulation within host tissues. However, it is yet to be determined whether these compounds interfere with SgRNA synthesis or directly target viral genomic RNA, thereby disrupting viral replication and subsequent protein synthesis processes.

#### 4.1.2. Suppression of Viral Protein Synthesis

In addition to targeting genomic RNA and lowering transcript levels, anti-TMV metabolites hinder TMV infection by selectively suppressing viral protein synthesis. The α-, β-Cembratriene-diols selectively inhibit CP biosynthesis but do not affect CP transcript levels in control and treated Nicotiana plants during qRT-PCR-based investigations [49]. A similar mechanistic resemblance was reported for Atisine-type diterpene alkaloids isolated from *Spiraea japonica* [41]. These compounds markedly reduced TMV CP accumulation in Western blot assays, whereas CP transcript levels remained unchanged between treated and control plants, suggesting selective inhibition at the translational or post-translational levels.

In addition, CP suppression-mediated anti-TMV mechanism was demonstrated by three quassinoids (chaparrinone, glaucarubinone, and ailanthone) isolated from *Ailanthus altissima*. These compounds showed dose-dependent inhibition of TMV CP accumulation in the Western blot assay, higher concentrations produced progressively stronger inhibitory effects [42]. Together, these studies illustrated that suppression of viral CP synthesis is an important inhibitory mechanism owned by different natural and synthetic anti-TMV compounds.

#### 4.1.3. CP Binding and Interference with Virion Assembly

The CP is essential for virion assembly, genome protection, and infection initiation, which involves RNA coating/uncoating and cell-to-cell movement [71]. Several plant-derived compounds target TMV by specifically binding to CP subunits, causing structural changes in the protein’s 3D shape and disrupting its interaction with RNA molecules, resulting in decreased viral infectivity and systemic movement [47,53]. *Peganum nigellastrum*-derived Luotonin A and its synthetic derivatives exhibit potent anti-TMV and antifungal properties primarily by targeting CP function [47]. Molecular docking and 20S CP disk analysis indicated that these compounds bind directly to TMV CP via hydrogen bonding and hydrophobic interactions. This interaction does not cause CP degradation; instead, it induces aberrant polymerisation and conformational disruption of CP, thereby impairing its ability to assemble correctly around viral RNA.

In contrast, Camalexin, a phytoalexin originally identified from *Arabidopsis thaliana*, and its synthetic derivatives containing the camalexin scaffold employ a different CP-mediated assembly-inhibition approach [48]. These compounds target CP assembly by directly disrupting CP structure, leading to loss of virion integrity and reduced viral infectivity. Mechanistic investigations revealed that representative compound **5a** induces fusion and disintegration of 20S CP disk intermediates, resulting in a distorted CP conformation and interfering with its orderly polymerization around viral RNA, ultimately preventing proper virion assembly. Moreover, diterpenoid alkaloids isolated from Dendrobium findlayanum [53], including findlayine A and dendrofindline B, showed a curative inhibition rate of 38.6% against TMV in tobacco leaves using the half-leaf assay, comparable to the commercial antiviral agent ningnanmycin (43.1%). The qRT-PCR analysis revealed significant transcriptional repression of the viral CP gene, indicating interference with viral gene expression. Furthermore, molecular docking demonstrated strong binding affinity of both compounds for TMV-CP, with hydrogen bonding and hydrophobic interactions identified as the main stabilising forces. Compounds such as ferulic acid dimers and α-aminophosphonate derivatives exhibited similar binding patterns at the CP’s interaction site, disrupting subunit aggregation and virion formation in in vitro assays [49,50]. Overall, these findings reinforce the central role of TMV-CP as an antiviral target, although the precise molecular mechanisms linking CP binding to impaired virion assembly remain to be fully elucidated for some compounds.

#### 4.1.4. TMV Particle Disruption

Disruption of virion integrity is a direct antiviral mechanism in which antiviral agents compromise the structural stability of TMV particles, resulting in partial or complete virion disassembly. Disruption of TMV particles is a direct antiviral mechanism in which external agents destabilise CP–CP or CP–RNA integration, causing the virion to partially or completely disintegrate [44,45,46,47]. This structural damage impairs the virus’s ability to remain infectious, as fragmented particles cannot properly uncoat, replicate, or spread in the host tissues. Several plant-derived metabolites and their synthetic derivatives exhibit this mode of action by directly damaging TMV particles. For instance, *C. lasianthera*-derived flavonoid glycosides induce extensive virion fracture accompanied by particle aggregation [46]. TEM observations revealed that treated TMV particles fragmented into smaller pieces ranging from 10 to 250 nm in length. Similarly, alkaloids extracted from Chelidonium majus, particularly chelerythrine, exhibit similar virucidal activity by disrupting intact TMV particles into smaller fragments [44].

In contrast, Osthole, a potent coumarin, isolated from *C. monnieri*, appears to interfere with virion formation by suppressing CP accumulation [45]. Western blot analysis demonstrated a dose-dependent reduction in CP accumulation, with complete inhibition observed at 7 mg/mL. However, whether osthole functions by inhibiting CP synthesis or by disrupting the stereoscopic assembly of the virus remains unresolved. Nevertheless, the mechanism underlying viral particle destruction is a common strategy employed by plant-derived metabolites to combat TMV infection.

### 4.2. Induced Resistance Mechanisms

**Figure 3:**
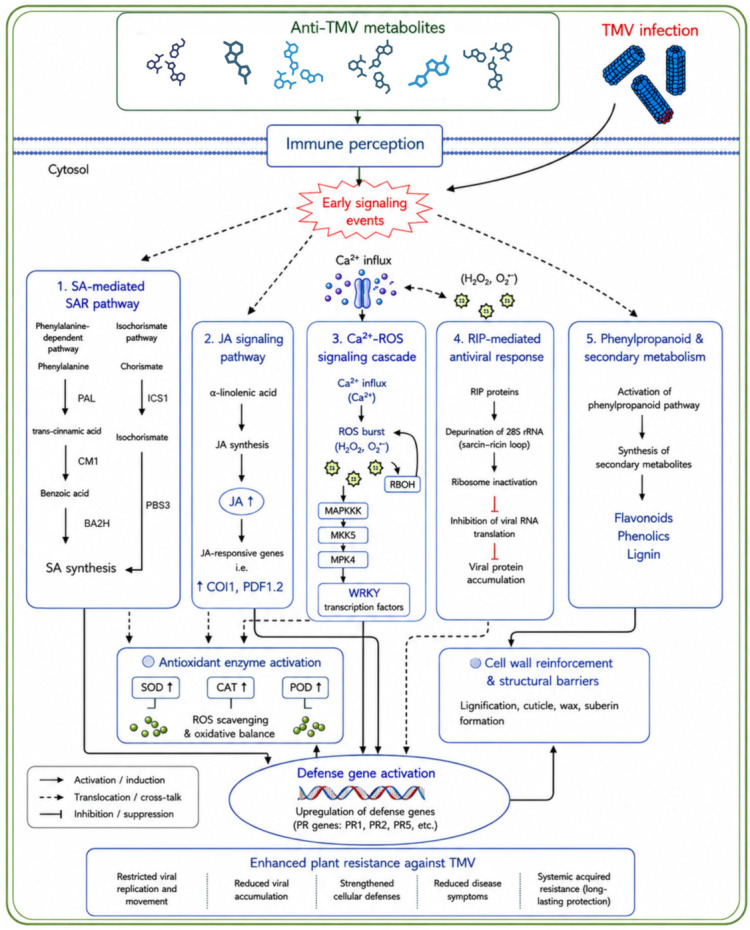
Schematic diagram of induced resistance mechanisms triggered by plant-derived anti-TMV metabolites. The picture was revised in BioRender https://BioRender.com (accessed on 6 July 2026). Created in BioRender. Siddiqui, H. (2026) https://BioRender.com/q2awptv (accessed on 6 July 2026).

Indirect antiviral activity is primarily achieved by activating and modulating host defence response mechanisms. Accumulated evidence from recent studies indicates that these indirect mechanisms can be broadly categorised into five major interconnected pathways (Figure 3): (i) salicylic acid (SA)-mediated systemic acquired resistance (SAR) [6], (ii) calcium (Ca^2+^)–reactive oxygen species (ROS)-mediated signalling [56], (iii) ribosome-inactivating protein (RIP)-mediated antiviral responses [63], and (iv) activation of phenylpropanoid-driven secondary metabolism [62]. These pathways do not function independently but instead form a coordinated, dynamic defence network that enhances plant resistance at multiple molecular and physiological levels.

#### 4.2.1. Salicylic Acid (SA)-Mediated Systemic Acquired Resistance

The SA-mediated induction of SAR mechanism serves as a central regulatory axis of antiviral immunity [72]. As per our current understanding, SA biosynthesis in plants occurs through two principal pathways: the phenylalanine-dependent pathway [73] and the isochorismate pathway [74]. In the phenylalanine route, phenylalanine is first converted into trans-cinnamic acid by phenylalanine ammonia lyase (PAL), followed by a series of reactions involving chorismate mutase 1 (CM1) that lead to the formation of benzoic acid, a key intermediate of this process. Benzoic acid is further hydroxylated to SA by benzoic acid 2-hydroxylase (BA2H), representing the final step in this pathway [75]. In parallel, the isochorismate pathway is mediated by isochorismate synthase (ICS1), which converts chorismate into isochorismate, followed by subsequent processing into PBS3 to generate SA [76]. Enhanced expression of PAL and CM1 promotes the accumulation of benzoic acid precursors, while increased BA2H activity facilitates their conversion into SA, collectively contributing to elevated SA levels during defence responses [75,76]. Compounds such as berberine [60], limonene [25], ursolic acid and 4-methoxycoumarin [6] have been shown to significantly elevate endogenous SA levels by upregulating the synthesis of PAL, ICS, and BA2H enzymes. Increased SA levels promote the expression of defence-responsive genes, like pathogenesis-related (PR) proteins (e.g., PR1, PR2, and PR5), by activating their central regulator NPR1 gene [77,78]. Exogenous application of Nicotiana tabacum isolated α(β)-cembratriene-diols activates SA-mediated induction of defence-associated genes (such as PR1, NPR1, and EDS1) [49]. Likewise, SA-deficient plants failed to provide resistance against TMV when treated with anti-TMV metabolite, 3-acetonyl-3-hydroxyoxindole (AHO) [61], emphasising the importance of this pathway [49]. Similarly, The jasmonic acid (JA) pathway also plays a role in antiviral defense, working synergistically or antagonistically with SA signaling to regulate plant immune responses [79]. For example, α(β)-cembratriene-diols simultaneously activate JA-responsive genes (COI1 and PDF1.2) along with SA markers [49], indicating a coordinated activation of both pathways that strengthens the plant’s defense. These cascades ultimately lead to the establishment of systemic acquired resistance (SAR), providing enduring and generalised protection against plant pathogens.

#### 4.2.2. Calcium (Ca^2+^)–Reactive Oxygen Species (ROS)-Mediated Signaling

The Ca^2+^–ROS cascade constitutes the earliest layer of the plant immune responses, acting as a rapid signal transduction mechanism upon elicitor recognition [80,81]. Compounds such as 4-hydroxychalcone [56] and the polysaccharide DNPE6(4) [54] have been shown to trigger transient calcium influx across cellular membranes, which in turn stimulates a burst of reactive oxygen species, particularly H_2_O_2_. This ROS accumulation functions not only as a direct antimicrobial agent but also as a secondary messenger that activates mitogen-activated protein kinase (MAPK) cascades, including MPK4 and MKK5, and transcription factors such as WRKY proteins, thereby initiating large-scale transcriptional reprogramming of defence genes [82]. Additionally, several SA-inducing compounds, including berberine and ursolic acid, contribute to ROS generation, highlighting the tight integration between ROS signalling and hormonal pathways [6,60]. This coordinated response strengthens cell wall barriers, promotes hypersensitive-like responses, and restricts viral replication and movement in host cells. Synthetic derivatives of cinnamic acid featuring glycoside scaffolds [62] trigger multiple defense-related pathways in *N. tabacum* plants, including MAPK signalling pathway, phenylpropanoid biosynthesis, glutathione metabolism, plant hormone signalling, and the biosynthesis of cutin, suberin, and waxes. Notably, among these derivatives, compound 8d significantly induces the activity of defence enzymes such as chitinase and β-1,3-glucanase, which triggers HR response in tobacco leaves during TMV infection [62,83]. These derivatives also regulate defence-related transcriptional and proteomic profiles, indicating involvement of JA-associated signalling and its interplay with secondary metabolic pathways [84,85].

#### 4.2.3. Ribosome-Inactivating Protein (RIP)-Mediated Antiviral Responses

Beyond classical immune-associated signalling cascades, ribosome-inactivating proteins (RIPs) provide a distinct and highly effective indirect antiviral mechanism that links host defence activation to direct inhibition of viral replication [64,86,87]. RIPs primarily depurinate a specific adenine residue in the highly conserved sarcin–ricin loop of 28S rRNA, irreversibly inactivating ribosomes and halting cellular translation [88]. During TMV infection, this activity preferentially limits viral RNA translation, given the virus’s high replication and translation demands, thereby reducing synthesis of viral proteins essential for replication, encapsidation, and cell-to-cell movement. CIP31, a member of the RIP family induced by cinchonaglycoside C [63], functions by depurinating ribosomal RNA, thereby arresting protein synthesis and specifically impairing production of the TMV CP, which is essential for viral assembly and systemic movement. Other RIPs, including pokeweed antiviral protein (PAP) [64], α-momorcharin (α-MMC) [86], MAP30 and luffin-α [87], not only dramatically reduce viral accumulation but also enhance expression of PR genes and antioxidant enzymes such as superoxide dismutase (SOD), catalase (CAT), and peroxidase (POD), thereby integrating direct antiviral effects with immune system activation.

#### 4.2.4. Activation of Phenylpropanoid-Driven Secondary Metabolism

The phenylpropanoid pathway and secondary metabolism play a crucial role in reinforcing plant defence by enhancing both structural and biochemical barriers [89]. Compounds such as ferulic acid–eugenol conjugates have been shown to increase PAL activity (a key enzyme in phenylpropanoid biosynthesis) and stimulate the synthesis of phenolic compounds, flavonoids, and lignin precursors, which contribute to cell wall strengthening and oxidative stress mitigation [50]. Myricetin derivatives reduce malondialdehyde (MDA) levels, a marker of lipid peroxidation, and increase antioxidant enzyme activity, thereby preserving cellular integrity during viral infections [51]. Eudesmanolides and polyphenolic glycosides such as swertisin and isoorientin similarly enhance PAL activity and antioxidant defences by increasing POD and SOD activity [59], further emphasising the importance of metabolic reprogramming in antiviral resistance. These findings collectively support a unified model of indirect antiviral defence against TMV infection. Early signalling events, driven by Ca^2+^ influx and a burst of ROS, trigger rapid responses that are then amplified via SA- and JA-dependent hormonal pathways. This results in strong transcriptional activation of defence genes, increased PR protein production, and elevated antioxidant levels. In contrast, RIPs directly suppress TMV replication at the translational level, while phenylpropanoid metabolism strengthens cellular structures and biochemical resilience against TMV infection [50,89].

## 5. Limitations and Future Directions

Although notable progress has been made in identifying plant-derived metabolites with anti-TMV activity, several key limitations continue to hinder their practical applications. A major challenge is the lack of extensive in vivo and field validation studies, as most research remains confined to laboratory tests or greenhouse experiments [41,46,50,53,63]. Moreover, TMV infects a broad range of economically important crops, including tobacco, tomato, pepper, cucumber, and numerous ornamental plants, but antiviral activity is most often evaluated in Nicotiana species or a limited number of model hosts [46,53,56]. This host bias limits the effectiveness of natural pesticides in real-world agricultural settings. To bridge this gap, multi-location field trials across diverse crop systems and environmental conditions are necessary to evaluate the efficacy, consistency, and environmental performance of promising plant-derived antiviral metabolites.

Another important research direction is to examine whether plant-derived metabolites can be used in combination with existing antiviral agents like ningnanmycin and ribavirin [21]. Such investigations may improve the overall effectiveness of antiviral agents, with reduced application rates and risk of resistance development. In addition, although many plant-derived metabolites have been observed to activate diverse defence-related pathways, it remains unclear whether these responses are specific to TMV or confer broader protection against other plant pathogens [39,54,56,64]. Moreover, the durability of induced resistance remains largely unexplored; how long the protective effects last after metabolite treatment needs to be assessed [51,52,54]. Time-course studies and pathogen-specificity assays are therefore required to characterise the durability and spectrum of induced resistance responses [90]. Such investigation may facilitate the development of broad-spectrum antiviral biopesticides with sustained protective effects. Despite increasing mechanistic investigations, the modes of action of many anti-TMV metabolites remain partly understood [18]. Anti-TMV metabolites have been observed to bind viral CP or disrupt viral RNA production; most evidence remains preliminary, based entirely on molecular docking predictions [51,52,62]. More rigorous mechanistic investigation employing molecular approaches and target validation experiments is needed to confirm these propositions. Another concern is that the antiviral efficacy is largely examined against limited TMV strains [45,56,63], efficacy against diverse TMV variants and related tobamoviruses is rarely assessed. Evaluating the broad-spectrum activity of plant-derived metabolites against diverse viral populations will be used to determine their practical utility under field conditions.

Finally, information on the toxicity and environmental safety of plant-derived antiviral metabolites remains limited. Most research focuses on their antiviral effectiveness, often neglecting their potential harm to crop plants, beneficial organisms, or non-target organisms, and soil microbial communities [18]. Although the natural metabolites are considered safe, their environmental persistence, degradation rate, and possible accumulation in soil and water need to be examined. In addition, these natural metabolites either have a direct influence on viral accumulation or, by activating host immunity, interfere with or alter plant normal metabolic processes, i.e., divert resources from growth and reproduction to antiviral defences, thereby negatively affecting biomass production and crop yield [14]. Such growth–defence trade-offs inflict a fitness cost on crop plants which may limit the potential utility of antiviral metabolites, particularly when long-term or repeated applications are required [91]. For example, pokeweed antiviral proteins PAPs exhibit strong anti-TMV activity through inactivation of host ribosomes, thereby suppressing the synthesis of viral proteins. However, these proteins are not entirely selective for viral components; their ribosome-inactivating activity also affects host cellular functions. Minor cell death symptoms were observed only in PAP-treated *N. benthamiana* [64], a potential fitness cost associated with enhanced antiviral defence. Hence, comprehensive toxicological, environmental and plant fitness evaluations are essential to confirm that these metabolites with a natural background are safe, sustainable, and appropriate for commercial-scale applications.

## 6. Conclusions

Plant-derived metabolites appear to be promising and sustainable antiviral agents against TMV, offering diverse mechanisms that target multiple stages of the viral infection cycle. Unlike conventional chemical pesticides, these natural compounds exhibit environmentally friendly characteristics, including biodegradability, reduced ecological toxicity, and lower risks of resistance development. Current evidence demonstrates that plant-derived metabolites and their synthetic derivatives can suppress TMV infection through both direct antiviral effects and indirect induction of host defence responses. Direct mechanisms include inhibition of viral RNA synthesis, suppression of CP accumulation, interference with virion assembly, and disruption of viral particle integrity. In parallel, indirect mechanisms involve activation of systemic acquired resistance, modulation of SA and JA mediated signaling pathways, induction of reactive oxygen species and calcium-dependent defence cascades, stimulation of ribosome-inactivating proteins, and enhancement of phenylpropanoid-associated secondary metabolism.

Recent advances in molecular biology, transcriptomics, proteomics, and computational approaches have significantly improved our understanding of the underlying antiviral mechanisms of these metabolites. Nevertheless, substantial gaps remain regarding their precise molecular targets, long-term efficacy, spectrum of activity, environmental safety, and practical applicability under field conditions. Future research should focus on integrating mechanistic investigations with translational applications, including toxicity assessment, field-scale validation, and the development of synergistic combinations with existing antiviral strategies. Expanding investigations toward broad-spectrum activity against diverse TMV strains and related tobamoviruses will also be essential for practical disease management. Overall, plant-derived metabolites highlight their importance toward sustainable and eco-friendly management of TMV and other viral diseases in crop plants.

## Figures and Tables

**Table 1:** Mechanisms of action of anti-TMV compounds, their source of origin and antiviral efficacy.

Direct Antiviral Mechanisms by Targeting Viral RNA			
Compound	Source	Antiviral Efficacy	Reference
Glaucogenin C, Cynatratoside A, Paniculatumoside C (Steroidal aglycone & glycosides)	*Strobilanthes cusia*	IC_50_: 0.017–0.025 μM MEC_50_: 0.001–0.002 μM	[37]
Antofine (Phenanthroindolizidine alkaloids)	Natural (Plant-derived)	86% inhibition IC_50_: 0.0044 μM	[38]
Suppressing the expression of viral proteins			
seco-pregnane C21 steroids and analogues (Steroidal glycosides)	*Cynanchum paniculatum*	IC_50_: 14.8–28.3 μg/mL	[39]
Tagitinin C (Sesquiterpenoid)	*Tithonia diversifolia*	Curative: 63% (100 μg/mL)	[40]
1β-Methoxydiversifolin-3-O-methyl ether (Sesquiterpenoid)	*Tithonia diversifolia*	Curative: 60% (100 μg/mL)	[40]
Atisine-type alkaloids (Diterpenoid alkaloids)	*Spiraea japonica*	64.45% inhibition (100 μg/mL)	[41]
Chaparrinone (Triterpenoids)	*Ailanthus altissima*	Inactivation: 53–56% Protective: 59–62% Curative: 40–46% (100 μg/mL)	[42]
Glaucarubinone (Triterpenoids)	*Ailanthus altissima*	IC_50_: 0.93 μM (leaf-disk) IC_50_: 7.35 μM (half-leaf)	[42]
Ailanthone (Triterpenoids)	*Ailanthus altissima*	IC_50_: 2.91 μM (leaf-disk) IC_50_: 7.92 μM (half-leaf)	[42]
N-methyl lycoricidine derivatives (Isoquinoline alkaloids)	Synthetic (Plant-sourced)	Inactivation: 72.57% Protective: 70.26% Curative: 61.97% (5000 μg/mL)	[43]
Disrupting TMV particle			
Chelerythrine (Isoquinoline Alkaloids)	*Chelidonium majus*	85–90% inhibition (500 μg/mL)	[44]
Osthole (Coumarins)	*Cnidium monnieri*	55–59% inhibition (500 μg/mL)	[45]
Flavonoid glycosides (Flavonoids)	*Clematis lasiandra*	Inactivation: 65–83% Protective: 58–59% Curative: 41–44% (500 μg/mL)	[46]
4-Methoxycoumarin (Coumarins)	Natural (Plant-derived)	Protective: 60–70% Curative: 50–60% Inactivation: 65–75% (5 μg/mL)	[6]
CP binding and virion assembly inhibition			
Luotonin A derivatives (Quinazolinone alkaloids)	Synthetic (Plant-sourced)	Curative: 53–61% (100 μg/mL)	[47]
Indole phytoalexin analogues (Indole alkaloids)	Synthetic (Plant-sourced)	78% inhibition (10 μg/mL)	[48]
Phenanthroindolizidine analogues (Phenanthroindolizidine alkaloids)	Synthetic (Plant-sourced)	80–91% inhibition IC_50_: 0.0037–0.0068 μM	[38]
α-aminophosphonate derivatives (Phosphorus–flavonoid hybrids)	Synthetic (Plant-sourced)	EC_50_: 356.7 mg/L	[49]
Ferulic acid Dimers (Phenylpropanoid)	Synthetic (Plant-sourced)	EC_50_: 63–85 μg/mL	[50]
Dual antiviral mechanisms by CP binding and inhibiting virion assembly, and inducing host immunity through selective regulation of defense enzymes			
Myricetin hybrid (Flavonols)	Synthetic (Plant-sourced)	EC_50_: 240 μg/mL (curative) EC_50_: 210 μg/mL (protective)	[51]
C-alkylated flavonoids (Flavones)	*Desmodium caudatum*	35.8–64.3% inhibition (50 μg/mL)	[52]
Dual antiviral mechanisms by CP binding and inhibiting virion assembly, and inducing the phenylpropanoid pathway mediated secondary metabolite synthesis			
Findlayine A, Dendrofindline B (Diterpenoid alkaloids)	*Dendrobium findlayanum*	38.6% inhibition (1000 μg/mL)	[53]
Host-mediated immune responses, by eliciting Ca^2+^ signalling pathways			
DNPE6(4) (Polysaccharide)	*Dendrobium nobile*	Protective: 69.9% ± 5.7% Curative: 23.6 ± 1.3 (125 μg/mL)	[54]
Eliciting Ca^2+^ signalling pathways and inducing SA-mediated SAR response			
Natural Polysaccharides	*Nicotiana tabacum*	Inactive & protective Inhibition: 80.40 -76.18% (500 μg/mL)	[55]
Inducing Ca^2+^–ROS signaling pathways			
4-Hydroxychalcone (Flavonoids)	Natural (Plant-derived)	Protective: 48.36% (1000 μM)	[56]
Activation of phenylpropanoid pathway and the synthesis of secondary metabolites			
Ferulic acid–eugenol conjugates (Phenylpropanoids)	Synthetic (Plant-sourced)	Inactivation: 57% Curative: 55% Protective: 53% (500 μg/mL)	[57]
Wedelolide C (Sesquiterpene)	*Wedelia trilobata*	Inhibition: 65.8% (10 μg/mL)	[58]
ROS-mediated signalling and activating the phenylpropanoid pathway mediated synthesis of secondary metabolites			
Swertisin, Comanthoside A and Isoorientin (Xanthone Glycosides-Polyphenols)	*Comastoma pedunculatum*	Inactivation: 51.65%, 45.66% and 12.85% Protective: 50.05%, 57.55% and 61.73% (100 μg/mL)	[59]
Inducing SA and JA pathways mediated SAR response			
α-, β-Cembratriene-diols (Diterpenoids)	*Nicotiana tabacum*	Protective: 71–73% (75 μM)	[49]
Inducing SA-mediated SAR response			
Ursolic acid (Triterpenoids)	Natural (Plant-derived)	Protective: 50–60% Curative: 40–50% Inactivation: 55–65% (5 μg/mL)	[6]
Berberine (Isoquinoline alkaloid)	*Coptis chinensis*	Protective: 63% Curative: 35% Inactivation: 14% (100 μg/mL)	[60]
Limonene (monoterpene)	Natural (Plant-derived)	Protective: 84.93% Curative: 58.89% (800 μg/mL)	[25]
Chelidonine (Isoquinoline alkaloids)	*Chelidonium majus*	Protective: 46–59% (100 μg/mL)	[44]
3-Acetonyl-3-hydroxyoxindole (Indole alkaloids)	*S* *trobilanthes cusia*	85% inhibition (500 nM)	[61]
Inducing SA-mediated SAR response and activating the phenylpropanoid pathway mediated synthesis of secondary metabolites			
Cinnamic acid glycoside (Phenylpropanoids)	Synthetic (Plant-sourced)	EC_50_: 130 μg/mL (protective)	[62]
RIP-mediated antiviral responses			
Cinchonaglycoside C (Steroidal glycosides)	*Strobilanthes cusia*	92% inhibition (0.5 μM)	[63]
Pokeweed antiviral protein (Ribosome-inactivating proteins)	Phytolacca americana	% antiviral activity not mentioned (100 μg/mL)	[64]

IC_50_: Half maximal inhibitory concentration (the concentration required to inhibit 50% of biological activity), MEC_50_: Median effective concentration (the concentration required to achieve 50% of the maximum effect), EC_50_: Half maximal effective concentration (the concentration required to produce 50% of the maximum effect), Inhibition: Suppression of viral infection or replication, Inactivation: Loss of viral infectivity, Curative: Ability of a metabolite to suppress viral infection when applied after virus inoculation, Protective: Ability of a metabolite to prevent viral infection when applied before virus inoculation, Synthetic (Plant-sourced): These compounds are chemically synthesized and their structures are derived or modified from naturally occurring plant metabolites. Natural (Plant-derived): Naturally occurring compounds isolated from plants, where the specific source plant is not mentioned.

## Data Availability

No new data were created or analyzed in this study. Data sharing is not applicable to this article.

## Abbreviations

The following abbreviations are used in this manuscript:

LRR	leucine-rich repeat
SAR	Systemic acquired resistance
RdRp	RNA-dependent RNA polymerase
RIPs	Ribosome-inactivating proteins
sgRNAs	Subgenomic RNAs
MP	Movement protein
CP	Coat protein
TEM	Transmission electron microscopy
ROS	Reactive oxygen species
CM1	Chorismate mutase 1
PAL	Phenylalanine ammonia lyase
BA2H	Benzoic acid 2-hydroxylase
PR	Pathogenesis-related
AHO	3-acetonyl-3-hydroxyoxindole
NPR1	Natriuretic peptide receptor 1
EDS1	Enhanced disease susceptibility 1
COI1	Coronatine-Insensitive 1
MAPK	Mitogen-activated protein kinase
PAP	Pokeweed antiviral protein
SOD	Superoxide dismutase
POD	Peroxidase
CAT	Catalase
